# Genome Characterization of *Stelechocarpus burahol* (Blume) Hook.f. & Thomson “Kepel” and Exploration of Phytochemicals from Water and Ethanolic Extracts of Leaves and Fruits

**DOI:** 10.3390/foods14203569

**Published:** 2025-10-20

**Authors:** Onsaya Kerdto, Pimpisid Koonyosying, Narisara Paradee, Sunhawit Junrungsee, Nopphadol Chalortham, Pheravut Wongsawad, Artit Yawootti, Amorntip Wongmuangsinghanat, Somdet Srichairatanakool

**Affiliations:** 1 Department of Biochemistry, Faculty of Medicine, Chiang Mai University, Chiang Mai 50200, Thailand; onsaya35@gmail.com (O.K.); pimpisid.k@cmu.ac.th (P.K.); narisara.p@cmu.ac.th (N.P.); 2 Department of Surgery, Faculty of Medicine, Chiang Mai University, Chiang Mai 50200, Thailand; sunhawit.j@cmu.ac.th; 3 Department of Pharmaceutical Science, Faculty of Pharmacy, Chiang Mai University, Chiang Mai 50200, Thailand; nopphadol.chalortham@cmu.ac.th; 4 Department of Biology, Faculty of Science, Chiang Mai University, Chiang Mai 50200, Thailand; pheravut.wong@cmu.ac.th; 5 Department of Electrical Engineering, Faculty of Engineering, Rajamangala University of Technology Lanna, Chiang Mai 50300, Thailand; yartit@rmutl.ac.th; 6 Golden—T Siam Company Limited, Pai, Maehongsorn 58130, Thailand; cmsskdec@gmail.com

**Keywords:** *Stelechocarpus*, kepel, burahol, genome, phenolics, antioxidant

## Abstract

*Stelechocarpus burahol* (kepel) is valued for its aromatic fruits and medicinal leaves, yet its genomic and phytochemical features remain poorly characterized. This study estimated the nuclear DNA content of kepel leaves at 3.96 pg per haploid genome (genome size: 3873 Mbp) and comprehensively profiled their bioactive metabolites. Leaf extracts prepared with water and 70% ethanol, with or without pulsed electric field (PEF) treatment, were analyzed using HPLC-MS, UHPLC-QTOF-MS, HPLC-DAD, and GC-MS. Leaf extracts showed the highest phenolic and flavonoid contents, with PEF markedly improving ethanolic extraction efficiency. A total of 72 phenolics, 2 tocopherols, 3 tocotrienols, and several novel vitamin E derivatives were detected, alongside abundant catechins, tannic acid, and gallic acid. PEF significantly enhanced catechin recovery: catechin (C) increased from 153.7 to 846.8 mg/g and epicatechin (EC) from 338.2 to 921.4 mg/g in water extracts, while ethanolic extracts rose from 335.3 to 905.1 mg/g (C) and 245.0 to 616.9 mg/g (EC). Epigallocatechin 3-gallate (EGCG), absent in untreated leaves, reached 799.9 mg/g in water and 231.9 mg/g in ethanol extracts after PEF. In fruits, PEF reduced phenolic recovery in water extracts (C: 236.7 → 136.8 mg/g; EC: 135.4 → 118.2 mg/g; EGCG: 2892.2 mg/g → undetectable), but slightly improved ethanolic extracts (C: 237.8 → 289.4 mg/g). GC-MS identified 19 volatile compounds contributing to the fruit’s aroma. This work provides the first integrated report of kepel genome size and phytochemical composition, highlighting PEF as a promising strategy to enhance leaf catechin extraction and supporting kepel’s potential as a functional food and medicinal resource.

## 1. Introduction

*Stelechocarpus burahol* (Blume) Hook.f. & Thomson, known as Burahol and kepel, belongs to the Annonaceae family (https://www.ipni.org/n/urn:lsid:ipni.org:names:2139-1 accessed on 5 May 2025). It was first taxonomized as *Uvaria burahol* and had been published in the Flora van Nederlandsch Indie, Collation i:94 (1855). Heusden previously published descriptions of the general features of the kepel plant, including trees up to 25 m high; glabrous and lenticel twigs; coriaceous, membranous, shiny elliptic to elliptic oblong (8–31 cm long, 2.5–9.5 cm wide) brown to green leaves; obovoid, subglobos, ellipsoid (30–70 mm long, 15–45 mm in diameter) glabrous or pubescent fruits with minute hairs, scurfy to verruculose; and horizontal, ellipsoid to broadly ellipsoid, flatly shaped seeds [1]. Due to traditional restrictions and policies pertaining to Indonesian Royal trees, kepel has been previously categorized as a rare plant in Indonesia [2]. Nowadays, kepel trees are cultivated and distributed in areas across South and Southeast Asia including Sumatra, Borneo, Java, Bali, Malaysia, Thailand, and Vietnam.

In addition to its cultural and botanical significance, kepel fruit is consumed both fresh and processed, valued for its sweet, aromatic flavor. Traditionally, Javanese royal families regarded it not only as a delicacy but also as a natural deodorant, as regular consumption was believed to impart a pleasant fragrance to the body [3]. Nutritionally, kepel fruit contains a variety of bioactive compounds such as flavonoids, phenolic acids, and essential fatty acid esters, many of which contribute to its antioxidant and anti-inflammatory properties [4]. The fruit also provides dietary fiber, vitamins, and minerals that support digestive health and overall well-being. These nutritional benefits, together with its ethnopharmacological applications, make kepel a promising candidate for functional food development and natural health products.

Importantly, species identification based on morphological characters can be utilized to precisely and scientifically recognize and establish the inter-species or intra-species diversity of *S. Burahol* [5]. Genome size and deoxyribonucleic acid (DNA) content represent key components of the unreplicated, basic, gametic chromosome set, providing a unifying measure that relates to important biological and ecological traits. This could ultimately lead to a better understanding of plant genome evolution [6,7]. The amount of DNA (*C*) and the number of complete sets of chromosomes (*N*) within a cell can change during the following events: a haploid gamete (1*C*, 1*N*), fertilization (2*C*, 2*N*), DNA replication (4*C*, 2*N*), and mitosis (2*C*, 2*N*). By using a molecular technique, plant genetics and genomics can be employed to utilize DNA barcoding that would lead to better accuracy and faster taxonomic identification [5]. Therein, inter sample sequence repeat (ISSR) markers and start codon targeted (SCoT) polymorphism have allowed researchers to investigate the genetic diversity and relationships among different cultivars [8]. Furthermore, polymerase chain reaction (PCR) has been utilized, along with specific primers, to amplify DNA fragments. Recently, Probojati and colleagues have identified a *S. burahol* DNA fragment length of 500–600 basepair (bp) and an abundance value of 63.9% with AT bases [5].

At present, kepel mesocarp can be cultured in Murashige and Skoog medium supplemented with picloram, wherein flavonoid compounds are produced during the 15-day log phase of growth [3]. Interestingly, ethanolic extract derived from *Stelechocarpus cauliflorus* or ngam ngo in Thai name (Family Annonaceae) *leaves* contained two dihydroflavonol glycosides, namely engeletin and astilbin, and these two compounds exert antioxidant, anti-inflammatory, and anti-glycation effects [9,10]. Conventional extraction methods (e.g., solid-liquid, maceration, enzyme/chemical hydrolysis, and Soxhlet extraction), as well as certain green techniques (e.g., pressurized fluid, supercritical fluid, freeze-thawing, ultrasonication, microwave, and pulsed electric field (PEF) extraction) have been used to achieve high-value ingredients, while valorization can maximize the extraction yield and enhance any nutritional, biological, and pharmacological properties [11,12]. The colorimetric Folin–Ciocalteu method was used for the determination of total phenolic content (TPC), while aluminum chloride-based assay was used for determination of total flavonoid content (TFC) [13,14]. In addition, headspace-solid phase microextraction (HS-SPME), followed by gas chromatography (GC)/mass spectrometry (MS), was used to effectively characterize the volatile organic substances of the entire fruits [15]. Likewise, polar phytochemicals can be quantified using a powerful form of high-performance liquid chromatography (HPLC) coupled with an ultraviolet-visible (UV-Vis), photodiode array (PDA), MS, or tandem MS/MS detector(s). This was accomplished through automated matching of the results of the UV spectra to the authentic standards of the in-house library, thereby authenticating the identity [16]. An ultrahigh performance liquid chromatography-electrospray ionization-quadrupole time-of-flight-mass spectrometry (UHPLC-ESI-QTOF-MS) machine can provide comprehensive profiling of many phytochemicals of plant extracts [17]. Moreover, headspace solid-phase microextraction gas chromatography-mass spectrometry (HS-SPME-GC-MS) is a nondestructive, solvent-free method that uses a thermal-regulated HS above a sample to extract volatile compounds onto a stationary adsorbent coated-fiber combined with GC for separation and MS for identification [18]. Furthermore, PEF pretreatment can improve the conventional 50% ethanol extraction method to achieve 91.6% increased TPC values, which correlated with the antioxidant activity recorded from olive pomace [19]. Importantly, the PEF technique has been successfully applied for the enhancement of polyphenol extraction from fresh food products and by-products [20].

A recent study has revealed that the methanolic extract of kepel fruits contains dodecanoic acid-propanetriyl ester; its methanol peel extract contains hexadecanoic acid methyl ester, hexamethyl tetracosahexaene and dodecanoic acid-propanetriyl ester; its ethyl acetate peel extract contains dodecanoic acid-propanetriyl ester; and its ethyl acetate fruit extract contains pelargonidin-malonylrhamnoside, 8-epiiridodial glucoside tetraacetate, epigallocatechin 3-gallate (EGCG), 5-octadecenal, 1,5-dicaffeoylquinic acid, 1,6-di-O-galloylglucose, and luteolin 7-O-glucoside. Notably, all the extracts exerted strong antioxidant activity [4]. Not only kepel fruits but also kepel leaves exert important nutritional and ethnopharmacological effects, including antioxidant, anti-hyperuricemic, inhibitory xanthine oxidase and cyclooxygenase, and anti-microbial activities [3]. Thus, this study aimed to investigate DNA content and size, and to identify the chemical compositions of the water and ethanolic extracts obtained from kepel leaves and fruits with and without with the use of the PEF.

## 2. Materials and Methods

### 2.1. Chemicals and Reagents

PCR master mix reagent was purchased from iNtRON Biotechnology Company, Yeonggi-do, Republic of Korea. NucleoSpin filters, spin column, and Plant II reagent kit including lysis buffers PL1 and PL2, precipitation buffer PL3, PCR buffer, washing buffers PW1 and PW2, elution buffer PE, and ribonuclease A (RNase A) were obtained from Macherey-Nagel GmbH & Co. KG, Duren, Germany. Nucleic acid staining dye “SafeView^TM^ FireRed dye” was purchased from Applied Biological Materials Inc., Richmond, BC, Canada. Agarose powder (Product number A9539, a low electroendosmosis value of 0.09–0.13), 50–5000 bp DNA marker (Catalogue number P9577), Folin–Ciocalteu reagent, aluminum chloride, sodium carbonate, potassium chloride, sodium acetate, and Tris-borate-ethylenediamine tetraacetate (TBE) were purchased from Sigma-Aldrich Chemicals Company Limited, Saint Louis, MO, USA. DNA dye (6X Loading ViSafe Gel Green dye, Catalogue Number: SD0101) was obtained from Vivantis Technologies, Selangor, Malaysia. Authentic standards, including EGCG, catechin (C), epicatechin (EC), gallic acid (GA) and quercetin (Q) were also purchased from Sigma-Aldrich Chemicals Company. Solvents, including acetonitrile, ethanol, ethyl acetate, methanol, and formic acid, were of HPLC or the highest-pure grade.

### 2.2. Kepel Plants

Kepel (*S. burahol*) trees were planted in the Demonstration Fields at Ban Pang Khum, Tambol Yangmin, Amphur Samerng, Chiang Mai Province, Thailand, at an altitude of 14,000 m. The crop, including stalks, leaves, flowers, and fruits, aged 8 years and 1 month, was harvested and sent for botanical identification, genome analysis, and chemical composition analysis, as will be described below.

### 2.3. Botanical Identification and Description

The plant was then described and identified according to its taxonomic aspects using standard procedures at the Institute of Herbal Medicine, Department of Medical Science, Ministry of Public Health, Nonthaburi, Thailand. Additionally, specimens were registered in the Herbarium, Faculty of Pharmacy, Chiang Mai University. Chiang Mai, Thailand.

### 2.4. Molecular Fingerprinting Using Inter-Simple Sequence Repeats

The ISSR-PCR is used to produce multilocus genetic markers based on length variations between microsatellites and valuable for investigating taxonomy, genetic diversity and phylogenetics in plants and fungi. ISSRs are DNA segments with different sizes (100–3000 bp) that can be amplified using PCR with microsatellite core sequences as primers as following.

#### 2.4.1. DNA Isolation

Firstly, 100 mg of fresh leaves of kepel, and Drawf Ylang-Ylang (*Cananga fruticose*) as a reference, were placed in a mortar and liquid nitrogen was poured over the samples. They were then crushed into a fine powder using a grinder. The pulverized samples were stored in 1.5 mL microcentrifuge tubes and DNA was extracted with a NucleoSpin Plant II reagent kit according to the manufacturer’s instructions. Firstly, 400 μL of a mixture of both lysis buffers PL1 and PL2 (1:1 by volume), and 10 μL of RNase A, were added to the powdered leaf samples. The mixture was then incubated in a 65 °C hot water bath for 30 min. Secondly, 1.5 mL of the cell lysate was transferred to a collection tube containing a NucleoSpin Filter and centrifuged at 11,000× *g* for 2 min. Thirdly, the filtrate was mixed with 450 μL of PCR PL3 buffer to precipitate DNA. Subsequently, the DNA was dissolved in 700 μL of buffer PW1, loaded onto a collection tube inserted with a NucleoSpin column (Silica membrane, dimension of 235 mm × 161 mm × 77 mm, binding capacity of 50 μg DNA with 50–50,000 bp size), and centrifuged at 11,000× *g* for 1 min. Fourthly, the DNA adsorbed onto the column was washed twice with 700 μL of buffer PW2 and eluted with 50 μL of buffer PE that had been prewarmed to 65 °C and subjected it to centrifugation at 11,000× *g* for 5 min twice. Finally, the pooled eluents were employed to determine the DNA amounts using a NanoDrop microvolume spectrophotometer (BioDrop Touch Duo, Product Number: 80-3006-61, Serial Number: BD1361, Cambridge, UK).

#### 2.4.2. PCR Method

The obtained genomic DNA extract was amplified using ten ISSR-labeled primers (Table 1) according to the PCR protocol established by Sultana et al. [21]. A working PCR master mix reagent was prepared according to the prescribed procedure (Table 2) and placed in the PCR machine (LifePro Thermal Cycler, Model: TC-96/G/H(b)A, Serial Number: BYQ6063E-245, Hangzhou BIOER TECHNOLOGY Company Limited, Hangzhou, China) in order to initiate the reaction for 35 cycles (Table 3).

#### 2.4.3. Agarose Gel Electrophoresis

For preparation of 1.5% agarose gel, 0.75 mg of agarose was melted in a microwave for 2 min, mixed with 50 mL of 1× TBE buffer pH 8.3, and allowed to solidify at room temperature for 15 min. In the assay, 5 μL of PCR products obtained from KP and KS leaves, along with 50–5000 bp DNA marker (M) were gently mixed with 3 μL of non-toxic SafeView^TM^ FireRed dye (sensitivity limit: 0.1–0.3 ng of DNA per band) at room temperature for 30 min. The mixtures were then dropped into the agar wells and run at a potential difference of 100 volts with constant current for 40 min. Finally, the gel was exposed to a UV-Transilluminator (Model: BECXF-20.M V1, Serial Number: 10 102612, VILBER LOURMAT Technologies, Marne-la-Vallée Cedex 3, Rue de Lamirault, Collégien, France), viewed for red fluorescence intensity (excitation wavelength of 540 nm and emission wavelength of 630 nm) of DNA bands, and the images were photographed for data analysis. The FI of DNA fragments from PCR product were compared against those of DNA standards or ladder and calculated their DNA contents per sample. In calculation, genome size was estimated using the conversion factor 1 pg of DNA ≈ 978 Mbp. DNA mass was measured by spectrophotometry, and genome size (Mbp) was calculated as DNA content (pg) × 978.

### 2.5. Preparation of Kepel Extracts

Firstly, 100 kg of fresh weight (FW) Kepel leaves were dried in a hot-air oven at 50–55 °C overnight, and the dry leaves (10 kg) were then ground using an electric blender (SharpThai Company, Limited, Bangkok, Thailand). Additionally, whole kepel fruits (10 kg) were chopped into small pieces, lyophilized with a freeze dryer (Labfreez Instruments Company, Limited, Changsha, Hunan, China), and ground using an electric blender. Afterward, 1 g of dry kepel leaves and fruits were extracted one time with 100 mL of DI water at 80 °C for 10 min or 100 mL of 70% (*v*/*v*) ethanol at room temperature overnight using the maceration method. After extraction, the samples were filtered through gauze sheets and Whatman No. 1 cellulose paper using a vacuum pump. The kepel leaf water extract (KLWE) and fruit water extract (KFWE) were dried using a freeze dryer. The filtrates of kepel leaf ethanolic extract (KLEE) and fruit ethanolic extract (KFEE) were then subjected to drying using a rotary evaporator at 40 rpm, 47.7–50.0 °C, and by applying the freeze-drying method, respectively [22].

### 2.6. PEF-Assisted Extractions

In assistance with PEF instrument (Heidolph Hei-VAP, Becthai Equipment & Chemical Company, Limited, Bangkok, Thailand), the generator of electric fields was powered by 220 VAC/50 Hz, and 500 watts. The chamber received a high voltage electric field (0–10 kV/cm and 1 μs) from the rotating gap switches. The coaxial-cylinder PEF chamber has a volume of 500 mL. The pulse repetition frequency (10 Hz), duration (1 μs), electric field strength (3 kV/cm), number of pulses (1200 pulses) were set, and the specific energy was accordingly calculated to be 2.61 kJ/kg of kepel powder [23,24]. In PEF treatment, dried leaf and fruit powder specimens (1 g) were mixed with DI water of 70% ethanol (100 mL) and extracted with PEF (^) at a field strength of 3 kV/cm, 5 min/cycle for 30 min at room temperature (25 ± 1 °C). The extraction chamber had a rectangular shape of 4.2 × 13.2 cm with electrodes spaced 2.0 cm apart. This was performed at a frequency of 50 Hz and the number of pulses recorded at 1200. The extraction chamber had a rectangular shape of 4.2 × 13.2 cm with electrodes spaced 2.0 cm apart. After extraction, the samples were filtered as described above. The kepel leaf water extract (KLWE^), leaf ethanolic extract (KLEE^), fruit water extracts (KFWE^) and fruit ethanolic extract (KFEE^) were dried using the methods as described above.

### 2.7. Colorimetric Determination of Chemical Compositions

#### 2.7.1. TPC

Kepel extract solution (100 µL) was mixed with 10% (*v*/*v*) Folin–Ciocalteu reagent (200 µL) and 10% (*w*/*v*) sodium carbonate (800 µL). The mixture was incubated at 25 °C for 30 min, and optical density (OD) was measured at a wavelength of 700 nm against a reagent blank using a double-beam UV-VIS spectrophotometer (Model UV-1800, Shimadzu Corporation, Kyoto, Japan). TPC was determined from a standard curve of GA and reported in terms of mg gallic acid equivalent (GAE)/g [13].

#### 2.7.2. TFC

Flavonoid content was determined using the colorimetric method based on the formation of a stable complex between aluminum ion and flavonoids [25]. Kepel extract solution (250 µL) was mixed with a chromogenic reagent containing 10% (*w*/*v*) aluminum chloride (50 µL), 1 M potassium acetate (50 µL), and DI (2.15 mL). The mixture was then incubated in the dark at 25 °C for 30 min, and OD was measured at a wavelength of 415 nm against a reagent blank using a UV-Vis spectrophotometer [14]. TFC was determined from a standard curve of Q and reported as mg quercetin equivalent/g (QE/g).

### 2.8. HPLC-ESI-MS Identification of Phenolic Compounds

Kepel extracts were analyzed for anthocyanins using the HPLC-MS method established by Cuyckens and colleagues with slight modifications [13,26]. The HPLC system (Agilent Technologies 1100 Series, Deutschland GmbH, Waldbronn, Germany) consisted of a quaternary pump (G1311A), an online vacuum degasser (G1322A), an autosampler (G1313A), a thermostated column compartment (G1316A), and a PDA detector (G1315A). The outlet of the PDA was coupled directly to the atmospheric pressure ESI interface of the MS detector (Agilent Technologies 1100 LC/MSD SL, Palo Alto, CA, USA) through a flow splitter (1:1). In terms of the analysis, kepel extracts were constituted in 1.0 mL of a mixture comprising solvent A (acetonitrile) and solvent B (10 mM formate buffer pH 4.0) (1:1, *v*/*v*), and then filtered through a syringe polytetrafluoroethylene (PTFE) membrane filter (25 mm diameter, 0.45-μm pore size, Corning Incorporation, Corning, NY, USA) before being analyzed. In the analysis, the sample (20 μL) was injected into the HPLC system, and the separation was carried out on a column (LiChroCART RP-18e, 150 mm × 4.6 mm, 5 µm particle size; Purospher STAR, Merck, Darmstadt, Germany) operated at 40 °C. Mobile phases A and B were run at a flow rate of 1.0 mL/min under the gradient program of 100% B (0% A) for an initial period of 5 min, 0–20% A from 5 to 10 min, 20% A from 10 to 20 min, 20–40% A from 20 to 60 min, 40% A for 3 min, and followed by an initial step of 100% B for 5 min. PDA detection was set at 270 nm. MS analysis was performed in positive ESI mode, and spectra were acquired within the mass-to-charge ratio (*m*/*z*) range from 100 to 700. For the single quadrupole MS system, the ESI energy was set at 70 eV, while the temperatures of the ion source and the interface were set at 150 °C and 230 °C, respectively. Nitrogen was used as the nebulizing, drying, and collision gas. The capillary temperature was set to 320 °C, the nebulizer pressure was set to 60 pounds•inch^2^, and the drying gas flow rate was set to 13 L/min. Capillary voltages were set to 3500 V (positive) and 150 V (negative). The oven temperature was programmed as follows: 80 °C (held for 3 min), ramped to 110 °C at 10 °C/min (held for 5 min), increased to 190 °C (held for 3 min), ramped to 220 °C at 10 °C/min (held for 4 min), and increased to 280 °C at 15 °C/min (held for 13 min). Accurate mass measurements were performed by employing the auto mass calibration method using an external mass calibration solution (ESI-L Low Concentration Tuning Mix; Agilent calibration solution B). Herein, the limit of detection (LOD), limit of quantitation (LOQ), and recovery value were found to be 0.5 mg/kg, 1.20 mg/kg, and 70–110%, respectively. The chromatographic and mass spectrometric analyses, and a prediction of the chemical formula, including the exact mass calculation, were performed by Mass Hunter software version B.04.00 built to 4.0.479.0 (Agilent Technologies, Deutschland GmbH, Waldbronn, Germany). In addition, MS data were searched for in published literature repositories.

### 2.9. UHPLC-ESI-QTOF-MS Analysis of Phenolic Compounds

Phenolic compositions of kepel extracts were analyzed using the comprehensive UHPLC-ESI-QTOF-MS method previously described by Hodgson and colleagues [27] with slight modifications [28]. The UHPLC-ESI-QTOF-MS system was composed of an UHPLC machine (Agilent 1260 Infinity II LC, Agilent Technologies, Inc., Santa Clara, CA, USA) equipped with an ESI-QTOF-MS. In the MS system, nitrogen gas nebulization was set at 45 pounds per inch^2^ with a flow rate of 5.0 L/min at 300 °C, the sheath gas was set at 11.0 L/min at 250 °C, and the capillary and nozzle voltage values were set at 3.5 kV and 500 V, respectively. A complete mass scan was conducted with *m*/*z* values ranging from 200 to 3200. All the operations, acquisitions, and analyses of the data were monitored using Agilent UHPLC-ESI-QTOF-MS MassHunter Acquisition Software Version B.04.00 “Find by Be” algorithm to generate a list of precise mass matches compounds. Peak identification was performed in positive modes using the library database, and the identification scores were further selected for characterization and *m*/*z* verification.

Lyophilized kepel extracts were reconstituted in a mixture of absolute methanol (100 µL) and Milli Q water (100 µL), ultrasonicated on an ice bath for 10 min, and centrifuged at 17,000× *g* at room temperature for 10 min. Afterward, the supernatant was passed through a syringe filter (cellulose ester type, 0.45 µm pore size, Merck Amicon filter, Sigma-Aldrich Chemical Company, Limited, Saint Louis, MO, USA). In the analysis, the filtrate (5 μL) was injected into the UHPLC system using an autosampler and fractionated on a column (InfinityLab Poroshell 120 EC octadecyl silane type, 2.1 mm × 100 mm, 2.7 µm particle size, Agilent Technologies Company, Santa Clara, CA, USA) that had been thermally regulated at 40 °C. It was then eluted in the linear gradient mode using mobile phase A (0.1% formic acid in DI) and mobile phase B (0.1% formic acid in acetonitrile) at a flow rate of 0.35 mL/min for 60 min. The timing program employed for gradient elution was as follows: 0 → 15 min; %A/B (100/0 → 90/10), 15 → 30 min; %A/B (90/10 → 40/60); 30 → 45 min; %A/B (40/60 → 10/90); and 45 → 60 min; %A/B (10/90 → 0/100). Peak identification was carried out in a positive mode using the library database, and the identification scores were sorted out for the purposes of characterization and *m*/*z* verification. For interpretation of MS/MS fragments, a stable molecular ion and characteristic analyte fragment ions were used to confirm structural features, differentiate between isomers, and assign Match Confidence Levels (MCL) beyond a single MS spectrum, following approach established by Schymanski et al. [29]. This system defines five confidential levels: Level 1, confirmed structure, supported by a reference standard with matching T_R_ and MS/MS spectrum; Level 2a, probable structure, based on a library spectrum; Level 2b, probable structure, supported by diagnostic evidence but lacking a reference spectrum); Level 3, tentative candidate, where multiple possible structures remain but are not fully confirmed; Level 4, unequivocal molecular formula assignment only; and Level 5, exact mass of interest only.

### 2.10. HPLC-DAD Quantification of Catechins

Briefly, the kepel extracts (10 mg) were reconstituted in 1 mL of DI water and passed through a syringe PTFE membrane filter. Then, 20 µL of the extract solution (1.0 g/100 mL) was injected into the HPLC-DAD system (Model 1290 Infinity II, Agilent Technologies, Inc., Santa Clara, CA, USA) and fractionated on a column (ODS type, 150 mm × 4.6 mm, 5 µm particle size, Agilent Technologies, Inc., Santa Clara, CA, USA) capped with a guard column (10 mm × 4.7 mm, 5 µm particle size, Agilent Technologies, Inc., Santa Clara, CA, USA). Individual catechins were eluted isocratically with mobile-phase solvent containing 0.05% H_2_SO_4_: acetonitrile: ethyl acetate (86:12:2, *v*/*v*/*v*) at a flow rate of 1.0 mL/min, and OD was detected at 280 nm with DAD. Standard C and EGCG at concentrations of 0–1 mg/mL were used to position the eluted EGCG peak, thereby generating a standard curve [30,31]. Accordingly, C, EC and EGCG concentrations of the kepel extracts were determined from the curves.

### 2.11. HS-SPME-GC-MS Analysis of Volatile Organic Compounds

The analysis was performed using the Agilent Instrument system (Agilent Technologies Company, Santa Clara, CA, USA). In principle, volatile organic compounds with small molecules by preparing the sample in the HS autosampler (Model Agilent PN 7697A), which involved the use of an oven heated to 70 °C. This would allow the analyte to evaporate into gas, move through and be captured by SPME resin before being eluted with a helium gas vehicle, fractionated on a stationary-phase column of GC machine (Model Agilent 7890B), and detected by an MS detector (Model Agilent 5977B) based on the *m*/*z* value. In the analysis, 1 μL of the sample obtained from HS-SPME was loaded onto a GC system equipped with a silica capillary column (30 m × 0.25 mm ID, 0.25 μm film thickness; Agilent 19091S-433) with carrier helium gas at a flow rate of 1.0 mL/min (injection temperature set from 60 °C for 2 min and then increased to 250 °C at a rate of 4 °C/min for 20 min), passed through an MS instrument set to scan at 70 and 230 electron volts over the *m*/*z* range of 29 to 350 amu, and the detected volatile compounds were characterized by comparison with the spectra of standard compounds [32]. For interpretation of MS/MS fragments, a stable molecular ion and characteristic analyte fragment ions were used to confirm structural features, differentiate between isomers, and assign MCL beyond a single MS spectrum, following approach established by Schymanski et al. [29]. This system defines five confidential levels: Level 1, confirmed structure, supported by a reference standard with matching T_R_ and MS/MS spectrum; Level 2a, probable structure, based on a library spectrum; Level 2b, probable structure, supported by diagnostic evidence but lacking a reference spectrum); Level 3, tentative candidate, where multiple possible structures remain but are not fully confirmed; Level 4, unequivocal molecular formula assignment only; and Level 5, exact mass of interest only.

### 2.12. Statistical Analysis

All quantitative experiments were performed in biological triplicates (*n* = 3) unless otherwise specified. Data were analyzed using the Statistical Package for the Social Sciences (SPSS) Statistics for Windows version 22 Program IBM Corporation, Armonk, NY, USA) and expressed as values of mean ± standard deviation (SD). For comparisons involving more than two groups, Significance was investigated using the one-way analysis of variance (ANOVA) test, followed by Tukey’s HSD post hoc test to correct for multiple comparisons. For pairwise comparisons, a two-tailed Student’s *t*-test was used. Where multiple hypotheses were tested simultaneously, false discovery rate correction was applied to minimize type I error. A threshold of *p* < 0.05 was considered statistically significant. When data did not meet the assumption of normal distribution, nonparametric tests were applied to determine significance.

## 3. Results

### 3.1. Taxonomy and Molecular Fingerprints of Kepel

**Figure 1:**
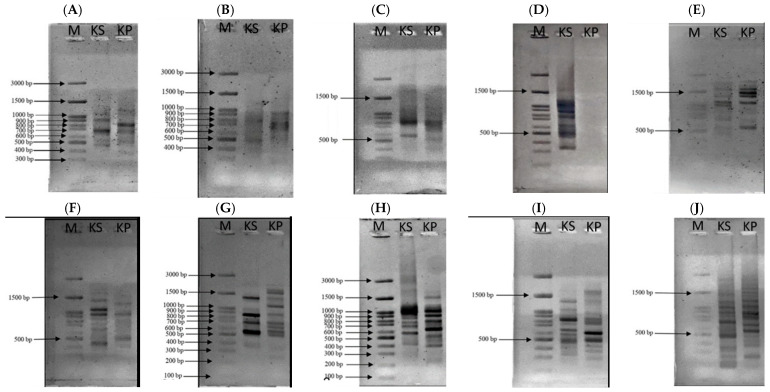
AGE analysis of DNA using primers UBC-807 (**A**), UBC-808 (**B**), UBC-809 (**C**), UBC-810 (**D**), UBC-811 (**E**), UBC-812 (**F**), UBC-834 (**G**), UBC-840 (**H**), UBC-841 (**I**), and UBC-842 (**J**). From the left, lane 1 = DNA marker, lane 2 = kepel leaves and lane 3 = Dwarf Ylang Ylang leaves.

The male flower samples of the kepel tree can be identified by plant taxonomy with the scientific name *S. burahol* (Blume) Hook.f. & Thomson Family: Annonaceae with a voucher number 0023315. According to the AGE method, ISSR markers and 10 tested primers were employed. DNA bands obtained from kepel leaves appeared at 300, 490, 600, 690, 900, and 1200 bp when using the UBC-807 primer, at 800 and 980 bp when using the UBC-808 primer, at 300, 490, 500, and 810 bp when using the UBC-809 primer, at 400, 500, 1400, 1500, 1600, and 2000 bp when using the primer UBC-811, at 330, 420, 500, 980, and 1300 bp when using the primer UBC-812, at 200, 500, 700, 800, 1000, 1400, and 1600 bp when using the primer UBC-834, at 400, 600, 890, 1100, 900, and 1200 bp when using the primer UBC-840, at 250, 280, 310, 400, 600, 820, 1000, 1200, and 1500 bp when using the primer UBC-841, and at 200, 300, 400, 590, 690, 700, 880, 900, 1000, 1300, 1500, 2000, and 2800 bp when using the primer UBC-842; nonetheless, no DNA bands were seen when the primer UBC-810 was used (Figure 1).

Genomic analysis of kepel leaves revealed that the nuclear DNA content was estimated to be 3.96 pg per haploid genome, which corresponds to a genome size of approximately 3873 Mbp (3.9 Gbp) [33].

### 3.2. Kepel Leaf and Fruit Extracts

As is shown in Table 4, the water and 70% ethanolic extracts of kepel leaves and fruits without or with PEF revealed different appearances, of which the ethanolic extracts had a more greas, intense brownness than the water extracts. Stoichiometrically, amounts of KLWE, KLEE, KFWE, KFEE, KLWE^, KLEE^, KFWE^, and KFEE^ were recorded at 11.8 ± 2.6, 8.8 ± 1.5, 16.8 ± 1.9, 10.4 ± 3.2, 11.8 ± 2.6, 8.8 ± 1.5, 16.8 ± 1.9, and 10.4 ± 3.2 g/100 g FW, respectively.

### 3.3. Total Phenolic and Flavonoid Contents in Kepel Extracts

**Figure 2:**
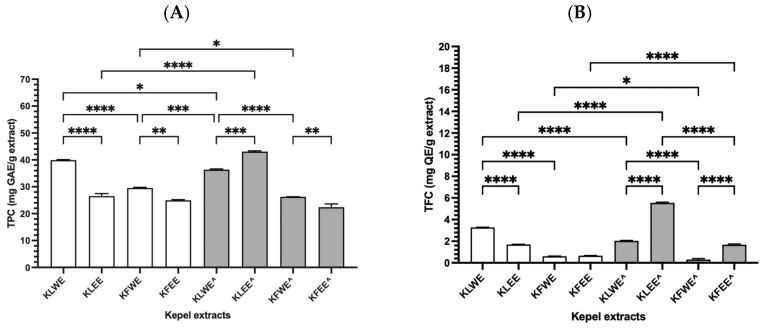
TPC (**A**) and TFC (**B**) values in the water and ethanolic extracts of kepel leaves and fruits with and without PEF (^) assistance. Data obtained from triplicate analysis are presented as mean ± SD values, for which * *p* < 0.05, ** *p* < 0.01, *** *p* < 0.005, and **** *p* < 0.001.

As can be seen in Figure 2A, TPC values were 39.92 ± 0.26, 26.53 ± 1.30, 29.54 ± 0.29, 24.97 ± 0.33 mg GAE/g) (*n* = 3) for KLWE, KLEE, KFWE and KFEE, respectively, using conventional extractions. Using PEF-assisted extractions, TPC values were 26.26 ± 0.07, 22.38 ± 1.25, 36.38 ± 0.33 and 43.08 ± 0.40 mg GAE/g (*n* = 3) for KLWE^, KLEE^, KFWE^ and KFEE^, respectively. In comparison, the TPC values in KLWE were significantly higher than KLEE (*p* < 0.001, Tukey’s HSD, *n* = 3), KFWE higher than KFEE (*p* < 0.01) The results suggest that the leaves contained more phenolic contents than the fruits. Notably, the water extraction method revealed greater amounts of phenolic contents than the ethanolic extraction method, while PEF enhanced the release of phenolic compounds. In comparison, TFC values obtained from three separate experiments were significantly higher in KLWE (3.28 ± 0.02 mg QE/g) than in KLEE (1.70 ± 0.02 mg QE/g) (*p* < 0.01), in KLEE^ (5.54 ± 0.09 mg QE/g) than in KLWE^ (2.03 ± 0.05 mg QE/g), and in KFEE^ (1.68 ± 0.09 mg QE/g) than in KFWE^ (0.30 ± 0.12 mg QE/g) (Figure 2B). Taken together, the results suggest that kepel leaves are richer in phenolics and flavonoids than kepel fruits. Water is better for phenolics whereas ethanol with PEF is better for flavonoids. PEF treatment significantly improves extraction efficiency.

### 3.4. HPLC-ESI-MS Profiles and Amounts of Phenolics in Kepel Extracts

**Figure 3:**
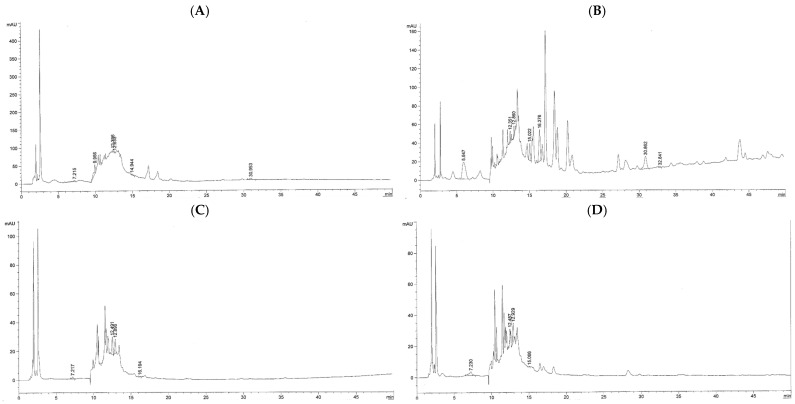
HPLC-ESI-MS profile of phenolic compounds obtained from KLWE (**A**), KLEE (**B**), KFWE (**C**), and KFEE (**D**).

The results of HPLC-ESI-MS analysis demonstrated that the water and ethanolic extracts obtained from kepel leaves and fruits contained gallic acid, catechins, and tannic acid; however, they were more abundant in KLEE and KFEE than in KLWE and KFWE. In addition, eriodyctoyl was found in 105.88 mg/kg of KLWE and 359.75 mg/kg of KLEE; isoquercetin in 257.26 mg/kg of KLEE and 1.88 mg/kg of KFWE; and quercetin in 29.13 mg/kg of KLEE. Nonetheless, hydroquinone, apigenin, and kaempferol were not detected in all the kepel extracts (Figure 3 and Table 5).

### 3.5. UHPLC-ESI-QTOF-MS Identification of the Phytochemicals of the Kepel Extracts

#### 3.5.1. Phenolic Compounds

**Figure 4:**
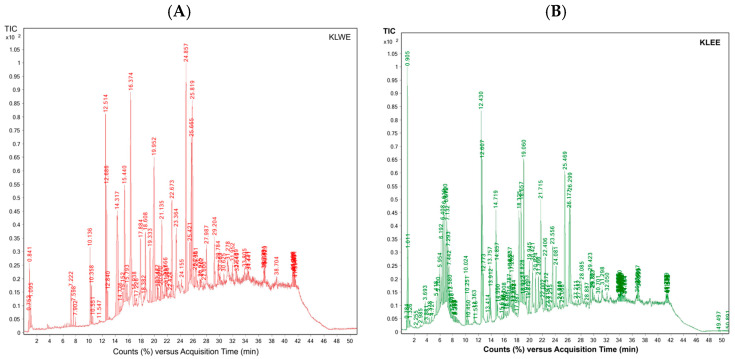

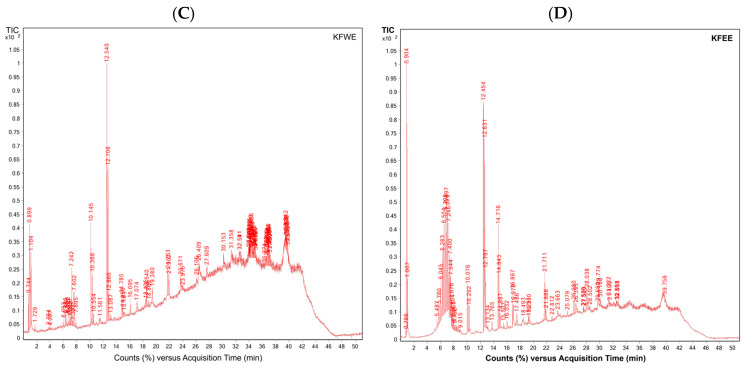
UHPLC-ESI-QTOF-MS profiles of phenolics, tocopherols, tocotrienols and their derivatives obtained from KLWE (**A**), KLEE (**B**), KFWE (**C**), and KFEE (**D**).

Compound identifications were based on accurate mass measurements and database/library searches using UHPLC-ESI-QTOF-MS. As authentic standards were not available for most compounds and MS/MS fragmentation data were not acquired, the results should be considered tentative or putative identifications. The findings (Figure 4) revealed a diverse profile of phenolic compounds in KLWE (A), KLEE (B), KFWE (C), and KFEE (D) samples. Correspondingly, Table 6 provides details of the tentatively identified compounds, including their retention time (minutes), observed and reference molecular weights (g/mol), mass error (ppm), and molecular formulas. The identified compounds can be categorized into five major groups as follows. Phenolic acids such as cinnamic acid, coumaric acid, ferulic acid, caffeic acid, chlorogenic acid, and sinapic acid were detected across all extracts. These were particularly abundant in ethanol-based extracts (Figure 4B,D), consistent with ethanol’s ability to solubilize medium-polarity phenolics. Flavonoids and their derivatives, including quercetin glycosides (e.g., quercetin 3-(2-glucosylrhamnoside), quercetin-3′-glucuronide), luteolin glycosides (e.g., luteolin 7-rhamnosylgalactoside, luteolin 5-malonylglucoside), and malvidin glycosides, were mainly present in the ethanol extracts. Their solubility pattern reflects the affinity of flavonoid aglycones and glycosides for alcoholic solvents. curcuminoids such as curcumin I, curcumin III, demethoxycurcumin, bisdemethoxycurcumin, hexahydrocurcumin, 5′-methoxycurcumin, and cyclocurcumin were concentrated in the ethanol extracts (KLEE and KFEE). This highlights the efficiency of ethanol in extracting hydrophobic phenolic compounds, which are poorly soluble in water. Gallic acid derivatives (e.g., gallic acid 3-O-galloylglucoside, glucogallic acid, ascorbyl-epigallocatechin gallate) were more frequently detected in water extracts (KLWE and KFWE; Figure 4A,C). Tannins are highly polar polyphenols, explaining their preferential extraction in aqueous media. A range of other bioactive compounds, including hydroxytyrosol glucoside, N-feruloyltyramine, urolithin A-glucuronide, carnosic acid, carnosol, and rosmanol derivatives were detected predominantly in ethanol extracts. These molecules are generally lipophilic, aligning with ethanol’s stronger extraction capacity for such compounds.

#### 3.5.2. Tocopherols and Tocotrienols in Kepel Leaf and Fruit Extracts

Interestingly, UHPLC-ESI-QTOF-MS analysis tentatively showed the presence of several tocopherols, tocotrienols, and related derivatives in kepel leaf extracts (Figure 4 and Table 7). α-Tocopherol (*m*/*z* 430.38) and δ-tocotrienol (*m*/*z* 396.30) were tentatively detected in the KFEE, while α-tocotrienol (*m*/*z* 424.33) was present in both KFWE and KFEE. In contrast, γ-tocotrienol (*m*/*z* 410.31) and tocopherol quinone (*m*/*z* 446.37) were tentatively identified only in the KLWE and KLEE. Tocopherol (*m*/*z* 416.36) was consistently observed across all extracts, indicating it as the predominant and most stable vitamin E derivative in kepel leaves. Tocopherol nicotinate (*m*/*z* 535.40) appeared solely in the KLEE. These findings suggest that fermentation and extraction solvents influence the selective enrichment or degradation of specific tocopherol and tocotrienol isomers in kepel leaf preparations.

#### 3.5.3. Amounts of C, EC and EGCG

**Figure 5:**
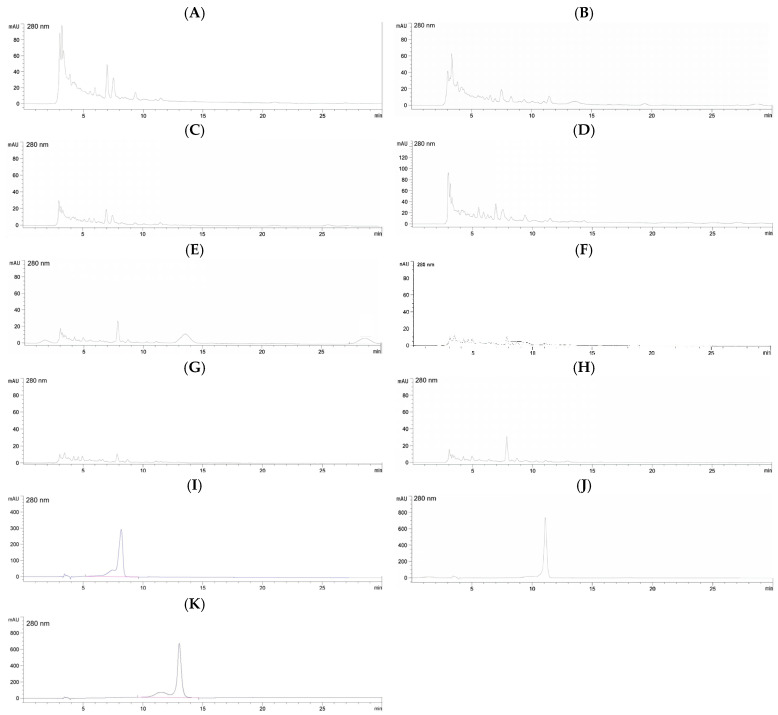
HPLC-DAD profiles of catechins in KLWE (**A**), KLWE^ (**B**), KLEE (**C**), KLEE^ (**D**), KFWE (**E**), KFWE^ (**F**), KFEE (**G**), KFEE^ (**H**) (5 mg/mL each), C (**I**), EC (**J**) and EGCG (**K**) (1 mg/mL each). Symbol: ^ = PEF.

Using HPLC-DAD with authentic standards, C, EC, and EGCG were confirmed and quantified in kepel extracts. The chromatograms (Figure 5A–H) and quantitative data (Table 8) clearly demonstrate that C, EC, and EGCG contents in *S. burahol* extracts are strongly influenced by solvent type and PEF treatment.

For leaf extracts, both KLWE and KLEE showed substantial increases in catechin and epicatechin levels after PEF. Catechin rose more than fivefold in water extracts (from 153.7 to 846.8 mg/g) and nearly tripled in ethanol extracts (from 335.3 to 905.1 mg/g). Similarly, epicatechin increased almost threefold in both solvents (KLWE: from 338.2 to 921.4 mg/g; KLEE: from 245.0 to 616.9 mg/g). Strikingly, EGCG, which was undetectable in untreated leaves, became abundant with PEF (799.9 mg/g in water extract and 231.9 mg/g in ethanolic extract). These results indicate that PEF disrupts leaf cell matrices, enhancing the release of flavan-3-ols and enabling detection of EGCG. In contrast, fruit extracts responded differently. Water extracts (KFWE) showed reductions in both C (from 236.7 to 136.8 mg/g) and EC (from 135.4 to 118.2 mg/g) after PEF, with EGCG completely lost (from 2892.2 mg/g to undetectable). Ethanolic extract (KFEE), however, showed modest improvements in C (from 237.8 to 289.4 mg/g) and EC (from 147.6 to 224.2 mg/g), but EGCG remained undetectable.

This suggests that PEF may degrade or destabilize certain catechins in fruit tissues, particularly EGCG, while improving recovery of other phenolics in ethanolic systems. Overall, the chromatographic profiles highlight a differential PEF effect: strongly positive in leaves, but solvent-dependent in fruits. These findings underline the importance of optimizing extraction conditions depending on plant tissue type and targeted phytochemicals.

#### 3.5.4. Volatile Organic Compounds

**Figure 6:**
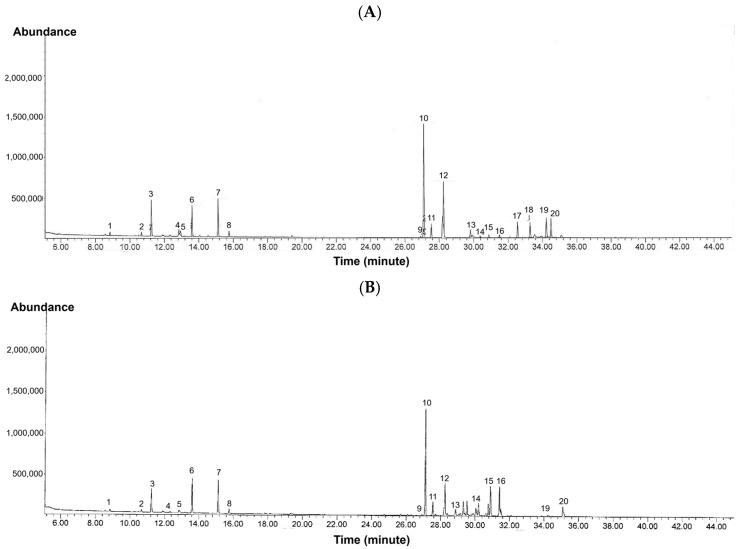
GC-MS profiles of volatile organic compounds in kepel fruits obtained using (**A**) HS-SPME and (**B**) ethanolic extraction.

GC-MS analysis of kepel fruits (Figure 6A,B) identified a diverse range of volatile organic compounds (Table 9). Regarding extraction principle and physicochemical properties of the volatiles HS-SPME is more sensitive to light, volatile monoterpenes while ethanolic extraction is more effective for heavier, less volatile, and more polar sesquiterpenes. Monoterpenes such as α-pinene, β-pinene, β-myrcene, limonene, and eucalyptol dominated the early eluting fractions, while sesquiterpenes including α-humulene, trans-β-caryophyllene, α-farnesene, and α-bisabolene were more prominent at higher retention times. Compounds were assigned MCL ranging from Level 2a (probable structure, library spectrum match) to Level 4 (unequivocal molecular formula only). The majority of identifications fell into Level 2a, supported by strong spectral matches (>90%), while a subset of sesquiterpenes and oxygenated derivatives such as nerolidol B, α-eudesmol, and bulnesol were assigned to Level 4 due to limited reference data. These findings indicate that both HS-SPME and ethanolic extraction capture complementary profiles of volatile constituents, with HS-SPME favoring more volatile monoterpenes and ethanolic extraction yielding higher molecular weight sesquiterpenes and alcohols. Both extraction approaches provided complementary qualitative profiles of the fruit’s volatile composition, highlighting the complexity of terpenoid constituents. Notably. Notably, some compounds appeared exclusively in either HS-SPME or ethanolic extracts. These differences are reported descriptively only, without inference of quantitative or statistical significance.

In addition, abundances of the volatiles per HS-SPME method versus ethanolic extraction) are expressed as relative % peak area of the total ion count (TIC). As the results, kepel fruit volatiles showed the presence of twenty compounds with varying relative abundances. The dominant constituent was trans-caryophyllene (30.82% of TIC), followed by α-humulene (10.30%), terpinolene (9.75%), and myrcene (8.70%). Other notable analytes included ocimene (7.63%), δ-cadinene (4.26%), γ-gurjunene (4.22%), β-eudesmol (6.05%), and bulnesol (5.17%). Compounds present in moderate to low proportions were α-pinene, β-pinene, limonene, eucalyptol, tricyclene, bergamotene isomers, farnesene, sesquiphellandrene, bisabolene, and nerolidol B, each representing less than 2% of the total ion current.

**Figure 7:**
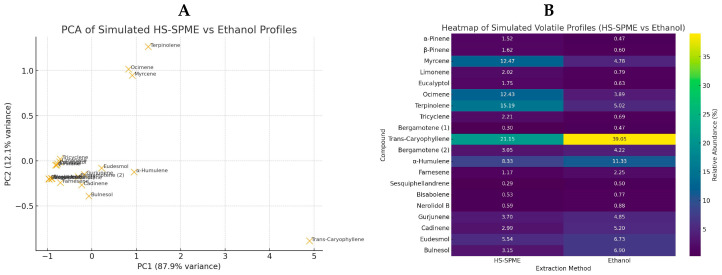
PCA (**A**) and heatmap (**B**) plots of volatile compounds detected in kepel fruits using simulated HS-SPME and ethanolic extraction profiles.

Moreover, the principal component analysis (PCA) and heatmap plots were created based on the simulated profiles of GC-MS and illustrated for comparing the HS-SPME versus ethanolic extraction. The PCA score plot (Figure 7A) shows a clear separation of compounds along PC1, with monoterpenes clustering toward HS-SPME (more volatile) and sesquiterpenes/oxygenated sesquiterpenes clustering toward ethanolic extraction (less volatile), suggesting the complementary selectivity of the two methods have been added. The heatmap plot (Figure 7B) highlights compound-specific enrichment patterns, with HS-SPME favoring light monoterpenes (such as α-pinene, myrcene, limonene and ocimene) and ethanol favoring heavier sesquiterpenes and oxygenated derivatives (such as trans-caryophyllene, α-humulene, eudesmol and bulnesol). Color intensity corresponds to relative abundance (% of TIC).

Overall, the volatile profile was characterized by a predominance of sesquiterpenes, especially trans-caryophyllene and α-humulene, supported by notable levels of monoterpenes such as myrcene, terpinolene, and ocimene. The presence of oxygenated sesquiterpenes, including β-eudesmol and bulnesol, further contributed to the complexity of the aroma, suggesting a sesquiterpene-abundant composition associated with woody, spicy, and herbal notes.

## 4. Discussion

Plant genome size refers to the total amount of DNA in a plant cell’s nucleus. It is usually measured in pg or bp. It can vary dramatically across plant species, with some having genomes more than 2000 times larger than others. In this case, it is important to recognize that approximately 90% of the nucleotide sequence can be repetitive. A unique DNA sequence is found only once per genome (or more than once, but in small numbers). In general, micro-satellite repeats consist of short repeats of one to six (and possibly as many as 10) bases and are approximately 16–25 nucleotides in length. In addition, the genomes of each organism contain varying numbers of tandem repeats distributed throughout the genome, resulting in a high degree of diversity, which make repeated nucleotide sequences useful as molecular markers in the study of plants and animals. Interestingly, ISSR markers use only one primer per reaction to amplify the DNA fragments located between two adjacent micro-satellite regions throughout the genomes. In contrast, random amplification of polymorphic DNA markers utilizes primers containing simple sequence repeats. Thus, ISSR markers are used to study the relationships of closely related organisms in populations and detect alleles specific to any organism. Regarding the advantages of employing ISSR markers, the method is not complicated, the results can be tested quickly, and the cost is relatively low [34]. Remarkably, ISSR markers have been chosen to analyze the apple genome and to possibly reveal polymorphisms in other plants [1,35,36,37].

Previous studies have identified universal DNA barcodes from chloroplast genomes such as ribulose-1,5-bisphosphate carboxylase/oxygenase large subunit, maturase K, transfer RNA-leucine (trnL), and transfer RNA-phenylalanine, which were used to optimize identification for the Family Annonaceae [38,39]. Recently, Probojati and colleagues used trnL intron sequences as a non-coding DNA barcode for the taxonomic identification of *S. burahol* and sister species complexes of the Annonaceae Family [5]. In addition, the genome sizes of other plants have been reported to be around 866.53 Mbp or 781.82 Mbp for *Orophea yunnanensis* and 44.7 Mbp for *Carica papaya* [40]. In this study, ISSR markers were employed. These markers are dominant, non-coding, non-quantitative, and are widely applied in genetic fingerprinting and diversity assessments. However, they do not provide comprehensive genome characterization since they yield only fragmentary information on genomic variation rather than quantitative or coding sequence data. As the results, the ISSR markers were successfully amplified and sequenced in *S. burahol* with a high-quality value. In this finding, the average DNA content was 3.96 pg per haploid genome and the genome size was 3873 Mbp. This study result has proved and confirmed the power of ISSR as a DNA barcode. It should, thus, be recommended for use in the identification of *S. burahol* and other species within the Annonaceae family.

By HPLC-MS analysis, ethyl acetate extract obtained from kepel fruit flesh was comprised of pelargonidin-malonylrhamnoside, 8-epiiridodial glucoside tetraacetate, EGCG, and 5-octadecenal, while ethyl acetate extract obtained from kepel fruit peel contained 1,5-dicaffeoylquinic acid, 1,6-di-O-galloylglucose, luteolin 7-O-glucoside, and 2-hydroxy-3-ethylidene-5-(methoxycarbonyl)-3,4-dihydro-2H-pyran-4-acetic acid 2-(3,4-dihydroxyphenyl)ethyl ester [4]. Moreover, antioxidant compounds, such as tocopherols, β-carotene, L-ascorbic acid, alkaloids, and saponins, as well as certain phenolics, namely flavonoids, cinnamic acid derivates, tannins, and coumarin, were reported [4]. Accordingly, these phytochemicals were found to exert antioxidant and antibacterial activities.

The present study has revealed that water and ethanolic extracts obtained from *S. burahol* leaves and fruits were abundant with phenolic and flavonoid contents such as catechins (HPLC-DAD analysis). Herein, the PEF operation significantly enhanced the ethanolic extraction of the phenolics and the flavonoids obtained from kepel leaves and fruits, but it did not influence the water extraction process. Notably, flavonoids are produced from *S. burahol* cultures in the log phase, for which the maximum production was reported on the 15th day [41]. Specifically, we highlight that while genera such as *Annona*, *Polyalthia*, and *Uvaria* are rich in flavonoids, phenolic acids, and acetogenins, *S. burahol* appears to be particularly abundant in polyphenols and flavonoids. This distinctive profile may account for its strong antioxidant activity and suggests complementary health benefits compared to its relatives

In our study, a comprehensive UHPLC-ESI-QTOF-MS technique could tentatively identify at least 72 phenolics and glycosides; α- and β-tocopherols; α- and δ-tocotrienols; γ-tocotrienol; α-tocopherolquinone; β-tocopherol; and α-tocopherol nicotinate in kepel leaves and fruits. In the findings, a broad spectrum of phenolic compounds across the four tested extracts (KLWE, KLEE, KFWE, KFEE). The tentative identifications encompassed phenolic acids, flavonoids, curcuminoids, tannins, and other bioactive phenolics, each showing distinct distribution patterns depending on the solvent used for extraction. The extraction solvent played a decisive role in shaping the phenolic composition. Water extracts (KLWE and KFWE) were particularly enriched in polar phenolic acids (e.g., cinnamic acid, caffeic acid, chlorogenic acid) and tannins/ellagitannins (e.g., gallic acid derivatives), consistently with previous reports highlighting the high polarity and water solubility of hydroxybenzoic and hydroxycinnamic acids as well as condensed tannins [42]. Conversely, ethanol extracts (KLEE and KFEE) exhibited a more complex profile dominated by curcuminoids, flavonoid glycosides, and diterpenoid phenolics, reflecting ethanol’s ability to dissolve medium- to low-polarity compounds and suggesting that the polarity index of phenolic compounds strongly dictates their extractability. Additionally, curcumin and its analogs have repeatedly been reported as predominant in ethanolic extracts of *Curcuma* spp. [43], while chlorogenic acid and gallic acid derivatives are often abundant in aqueous plant extracts [44].

The HPLC-DAD results reveal distinct solvent- and tissue-dependent effects of PEF on catechin recovery from S. burahol. In leaves, PEF markedly enhanced extraction of C, EC, and enabled the detection of EGCG, which was absent in untreated controls. This strong positive effect is likely due to electroporation-induced disruption of leaf cell walls and vacuoles, facilitating solvent penetration and metabolite release. Similar improvements in flavonoid recovery have been reported in other plant matrices subjected to PEF, underscoring its utility as a non-thermal intensification technique. Conversely, in fruits, PEF treatment did not enhance C recovery in water extracts and instead caused sharp reductions, particularly the complete loss of EGCG. Possibly, fruit tissues, richer in sugars, organic acids, and oxidative enzymes, may foster catechin degradation under PEF-induced stress. EGCG is known to be highly unstable and prone to oxidation, which could account for its disappearance post-treatment. The modest improvements observed in ethanolic fruit extracts suggest that ethanol may provide partial stabilization of phenolics against degradation, though not sufficient to preserve EGCG. The contrasting outcomes highlight the complexity of PEF-assisted extraction. In leaves, PEF acts as an effective enhancer of phenolic recovery, whereas in fruits its impact is solvent-dependent and may even be detrimental to certain labile compounds. This dual response underscores the need for tailored optimization of PEF parameters and solvent systems for different plant tissues. Importantly, the discovery of PEF-released EGCG in leaves expands the known phytochemical profile of kepel and supports its potential as a functional food resource.

The HS-SPME-GC-MS platform is a powerful analytical method developed for extracting and identifying volatile organic compounds. It offers the advantages of easy solvent-free sample preparation, simple manipulation, high purity of volatile analytes in the extracts, potential automation, and enhanced sensitivity. HS-SPME-GC-MS analysis demonstrated that pulp methanolic extracts contained dodecanoic acid-propanetriyl ester (lauric acid ester); the peel methanolic extract contained hexadecanoic acid methyl ester (or methyl palmitate), hexamethyl-tetracosahexaene (or squalene) and dodecanoic acid-propanetriyl ester; and the peel ethyl acetate extract contained dodecanoic acid-propanetriyl ester [4]. Wang and colleagues have reported a total of 348 volatile compounds. These included 60 esters, 55 alkenes, 45 aldehydes, 45 ketones, 37 alcohols, 20 aromatic hydrocarbons, and 66 other compounds that were identified in citrus blend black tea by using HS-SPME-GC-MS [45]. In contrast, our findings have revealed at least 20 volatile compounds from ripe kepel fruits. Regarding extraction principle and physicochemical properties of the volatiles HS-SPME is more sensitive to light, volatile monoterpenes while ethanolic extraction is more effective for heavier, less volatile, and more polar sesquiterpenes. In this study, the findings revealed that both HS-SPME and ethanolic extraction capture complementary profiles of volatile constituents, with HS-SPME favoring more volatile monoterpenes and ethanolic extraction yielding higher molecular weight sesquiterpenes and alcohols. Regarding PCA and heatmap plots, HS-SPME emphasizes the fresh, herbal aroma from monoterpenes while ethanolic extraction highlights the woody, spicy notes from sesquiterpenes, providing complementary volatile profiles of kepel fruits.

Beneficially, the sophisticated analytical instruments used in this study disclose new polar phenolic constituents in kepel leaves. The kepel leaves were found to possess herbal properties and contain volatile organic constituents, thereby potentially offering aromatic and nutraceutical benefits. Another key aspect of our findings is the role of PEF in enhancing phytochemical extraction. PEF is a nonthermal, environmentally friendly technology that permeabilizes plant cell membranes, thereby facilitating the release of phenolics, flavonoids, and other intracellular compounds. This approach preserves thermolabile bioactivities, maintains antioxidant activity, and increases overall extraction yields compared with conventional methods. In terms of the relevant limitations, there are not many kepel trees in Thailand. Moreover, kepel fruits are very expensive, as they are considered a seasonal fruit. Therefore, kepel seedlings and kepel tissue cultures need to be propagated within the country, so that the biological and pharmacological activities of kepel plants can be urgently investigated.

Collectively, we elaborated on the role of different primers (ISSR and SCoT), highlighting how their amplification of distinct genomic regions provides complementary insights into the genetic diversity of *S. burahol*. We also discussed the importance of integrating additional marker systems (e.g., SSRs, SNPs) in future work for higher resolution and validation. We strengthened our interpretation by emphasizing the biological relevance of the identified compound classes (flavonoids, phenolic acids, anthocyanins, fatty acid esters) and their contribution to the observed antioxidant activity. Notably, water and ethanol extractions yield complementary sets of phenolics. Water preferentially extracts highly polar compounds, while ethanol favors less polar, lipophilic bioactive phytochemicals such as curcuminoids and diterpenoids. However, the combined use of aqueous and ethanolic extractions may maximize recovery of bioactive compounds, enhancing both nutraceutical value and functional applications of the extracts. We also noted the potential synergistic effects of these compounds within the extracts. We acknowledged that some compounds were identified based on mass spectral libraries only. We have added a statement recommending validation with authentic analytical standards (e.g., EGCG, luteolin-7-O-glucoside, caffeoylquinic acid derivatives) in future studies to enhance confidence in compound identification and quantification.

## 5. Conclusions

Overall, the chemical compositions of the water and ethanol extracts can differ due to the differing polarity of the solvents, which can then affect the contents and antioxidant activity. Pulse electric field operation can enhance the efficiency of the solvent extractions of the flavonoids, but not the phenolics, that were obtained from kepel leaves and fruits. Among the phenolic constituents of kepel extracts, catechin, epicatechin, and epigallocatechin 3-gallate were confirmed and quantified using authentic standards, while the remaining compounds were tentatively identified by UHPLC-QTOF-MS. These findings highlight the rich phenolic composition of kepel, although further work using additional standards and MS/MS validation is needed to confirm the full phytochemical profile. Future studies should assess the extracts’ toxicities and anti-hepatic lipid deposition in cell cultures and in vivo models to provide confirmation.

## Figures and Tables

**Table 1:** ISSR-labeled primers used in this assay.

Primer Name	Nucleotide Sequence (5′ → 3′)	T_m_ (°C)	Primer Length (bp)
UBC-807	AGAGAGAGAGAGAGAGT	46.43	17
UBC-808	AGAGAGAGAGAGAGAGC	70.48	17
UBC-809	AGAGAGAGAGAGAGAGG	0.48	17
UBC-810	GAGAGAGAGAGAGAGAT	87.44	17
UBC-811	GAGAGAGAGAGAGAGAC	67.46	17
UBC-812	GAGAGAGAGAGAGAGAA	11.45	17
UBC-834	AGAGAGAGAGAGAGAGYT	74.48	18
UBC-840	GAGAGAGAGAGAGAGAYT	0.47	18
UBC-841	GAGAGAGAGAGAGAGAYC	46.48	18
UBC-842	GAGAGAGAGAGAGAGAYG	77.48	18

Abbreviations: bp = base-pair, T_m_ = melting temperature.

**Table 2:** Compositions of the PCR master mix reagent.

Reagents	Volume (μL)
DI water	7.11
10× PCR buffer	0.2
10 mM dNTPs	0.2
ISSR primer	0.2
i-TaqTM plus DNA polymerase	3.0
DNA template	0.2

Abbreviations: DI = deionized, DNA = deoxyribonucleic acid, dNTPs = deoxyribonucleotide triphosphates, ISSR = Inter-simple sequence repeat, PCR = polymerase chain reaction.

**Table 3:** PCR steps.

PCR Cycle	Temperature (°C)	Time (min)
Initial Denaturation	0.94	0.4
Denaturation	0.94	40.0
Annealing	0.48	45.0
Extension	0.72	0.02
Final extension	0.72	0.05
Parking	4.0	-

**Table 4:** Appearance and yields of water and ethanolic extracts obtained from kepel leaves and fruits with and without PEF(^). Amount of fresh, dry, and lyophilized kepel samples are reported in absolute values of g/100 g FW.

Sample	Fresh Weight (g)	Dry Weight (g)	Lyophilized Extract	Appearance	Yield (g)	Yield (%)
KL	107	11.81	KLWE	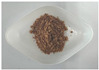	0.110	11.04
KL	51	10.56	KLEE	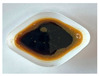	0.208	20.83
KF	42	13.66	KFWE	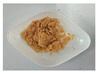	0.321	32.15
KF	43	12.09	KFEE	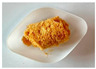	0.280	27.98
KL	50	9.98	KLWE^	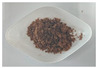	0.200	19.95
KL	50	3.75	KLEE^	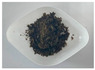	0.075	7.50
KF	50	6.55	KFWE^	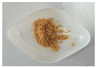	0.131	13.10
KF	50	6.45	KFEE^	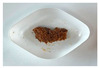	0.129	12.91

Abbreviations/symbols: KFEE = kepel fruit ethanolic extract, KFEE^ = kepel fruit ethanolic extract with PEF, KFWE = kepel fruit water extract, KFWE^ = kepel fruit water extract with PEF, KLEE = kepel leaf ethanolic extract, KLEE^ = kepel leaf ethanolic extract with PEF, KLWE = kepel leaf water extract, KLWE^ = kepel leaf water extract with PEF, PEF = pulse electric field, ^ = PEF.

**Table 5:** Absolute values of phenolic content obtained from KLWE, KLEE, KFWE, and KFEE samples assayed by the HPLC-ESI-MS method.

Peak	T_R_ (min)	Phenolics	KLWE	KLEE	KFWE	KFEE
1	4.2–5.6	Gallic acid (mg/kg)	52.44	375.65	13.41	63.92
2	12.5	Catechins (mg/kg)	136.78	271.28	384.85	141.25
3	12.8	Tannic acid (mg/kg)	7.60	139.60	187.72	345.91
4	15.0–15.6	Rutin (mg/kg)	44.76	189.31	ND	4.58
5	16.2	Isoquercetin (mg/kg)	ND	257.26	1.88	ND
6	23.8	Hydroquinine (mg/kg)	ND	ND	ND	ND
7	30.7	Eriodyctoyl (mg/kg)	105.88	359.75	ND	ND
8	33.1	Quercetin (mg/kg)	ND	29.13	ND	ND
9	41.2	Apigenin (mg/kg)	ND	ND	ND	ND
10	42.6	Kaemferol (mg/kg)	ND	ND	ND	ND

Abbreviation: ND = not detectable.

**Table 6:** Tentative existence of phenolic compounds in KLWE (A), KLEE (B), KFWE (C), and KFEE (D) assayed using UHPLC-ESI-QTOF-MS.

T_R_ (min)				Mass (g/mol)		Error	Molecular	Tentative Compounds
A	B	C	D	Reference	Observed	(ppm)	Formula	
0.824	0.861	0.855	0.864	358.090	358.089	−4.07	C_15_H_18_O_10_	Dihydrocaffeic acid 3-O-glucuronide
ND	0.861	ND	0.847	322.069	322.067	−4.82	C_15_H_14_O_8_	Gallocatechol
0.868	ND	ND	ND	368.126	368.126	0.05	C_21_H_20_O_6_	Cyclocurcumin
0.874	42.015	21.686	21.634	336.136	336.134	−6.31	C_21_H_20_O_4_	Curcumin III
ND	0.878	0.855	ND	372.106	372.104	−5.59	C_16_H_20_O_10_	Dihydroferulic acid 4-O-glucuronide
ND	1.315	0.888	ND	224.069	224.068	−2.48	C_11_H_12_O_5_	cis-Sinapic acid
ND	0.889	ND	ND	484.085	484.083	−4.28	C_20_H_20_O_14_	Gallic acid 3-O-(6-galloylglucoside)
ND	ND	0.910	ND	312.048	312.045	−8.63	C_13_H_12_O_9_	cis-Caffeoyl tartaric acid
ND	0.911	ND	ND	610.153	610.154	1.45	C_27_H_30_O_16_	Quercetin 3-(2-glucosylrhamnoside)
0.951	0.933	ND	0.913	148.052	148.052	−4.96	C_9_H_8_O_2_	Cinnamic acid
1.089	1.089	1.104	0.913	164.047	164.047	−4.43	C_9_H_8_O_3_	m-Coumaric acid
1.089	0.917	0.766	1.089	150.068	150.068	−1.51	C_9_H_10_O_2_	Hydrocinnamic acid
1.095	9.792	1.131	ND	206.094	206.093	−5.66	C_12_ H_14_O_3_	Ethyl p-methoxycinnamate
1.095	ND	1.098	ND	180.042	180.041	−5.81	C_9_H_8_O_4_	Caffeic acid
ND	ND	1.098	ND	194.058	194.057	−2.70	C_10_H_10_O_4_	Ferulic acid
1.543	0.928	1.729	1.765	190.099	190.099	−3.92	C_12_H_14_O_2_	3-Dimethylallyl-4-hydroxybenzaldehyde
2.726	0.944	3.509	2.981	313.131	313.130	−4.54	C_18_H_19_NO_4_	N-Feruloyltyramine
3.799	3.871	ND	ND	466.111	466.108	−7.78	C_21_H_22_O_12_	Epicatechin 7-O-glucuronide
3.816	3.870	ND	ND	484.085	484.083	−5.69	C_20_ H_20_O_14_	Gallic acid 3-O-(6-galloylglucoside)
5.740	5.667	ND	ND	594.159	594.155	−5.11	C_27_H_30_O_15_	Luteolin 7-rhamnosyl(1->6) galactoside
5.954	ND	ND	5.954	338.115	338.116	0.53	C_20_H_18_O_5_	Demethoxycurcumin
9.206	ND	ND	ND	396.157	396.154	−8.67	C_23_H_24_O_6_	Curcumin I
9.206	ND	ND	ND	374.173	374.172	−2.12	C_21_H_26_O_6_	Hexahydrocurcumin
11.578	11.578	10.694	ND	260.105	260.104	−2.09	C_15_H_16_O_4_	Dimethylallyl scopoletin
ND	13.962	1.331	ND	310.105	310.104	−5.40	C_15_H_18_O_7_	trans-Cinnamoyl-β-D-glucoside
ND	13.962	3.769	ND	331.082	331.081	−2.62	C_17_H_15_O_7_	Malvidin
ND	14.210	14.342	14.246	308.105	308.103	−4.70	C_19_H_16_O_4_	Bisdemethoxycurcumin
14.334	18.303	ND	ND	128.047	128.047	−1.91	C_6_H_8_O_3_	Dihydrophloroglucinol
14.559	ND	ND	ND	166.063	166.063	2.73	C_9_H_10_O_3_	Methyl 2-hydroxyphenylacetate
ND	15.057	ND	15.048	398.137	398.136	−1.75	C_22_H_22_O_7_	5′-Methoxycurcumin
15.290	13.375	ND	18.493	416.220	416.219	−2.42	C_24_H_32_O_6_	Deoxyschizandrin
15.594	15.538	15.598	15.534	314.115	314.114	−3.95	C_18_H_18_O_5_	2′-Hydroxyenterolactone
14.317	18.308	1.104	ND	152.047	152.047	−1.22	C_8_H_8_O_3_	2-Hydroxyphenylacetic acid
16.396	19.082	ND	ND	433.114	433.112	−4.65	C_21_H_21_O_10_	Isopeonidin 3-arabinoside
16.711	ND	ND	ND	404.074	404.076	3.19	C_19_H_16_O_10_	Urolithin A-3-O-glucuronide
17.976	ND	ND	ND	316.116	316.114	−5.49	C_14_H_20_O_8_	Hydroxytyrosol 1-O-glucoside
18.597	18.358	ND	17.304	360.194	360.195	4.20	C_21_H_28_O_5_	7-Methylrosmanol
18.641	14.017	ND	18.001	332.199	332.201	7.58	C_20_H_28_O_4_	Carnosic acid
18.664	20.470	ND	ND	346.178	346.180	6.10	C_20_H_26_O_5_	Epirosmanol
19.360	6.203	ND	ND	371.173	371.173	−2.17	C_21_H_25_NO_5_	3,4-Dimethoxyphenyl(ethyl)-3,4-dimethoxycinnamic acid amide
21.130	20.382	24.224	21.199	126.032	126.032	2.57	C_6_H_6_O_3_	Phloroglucinol
21.191	2.985	ND	3.485	313.131	313.132	2.66	C_18_H_19_NO_4_	N-Feruloyltyramine
21.329	15.145	ND	14.744	374.209	374.210	0.57	C_22_H_30_O_5_	11,12-Dimethylrosmanol
21.650	22.572	ND	ND	390.204	390.204	0.53	C_22_H_30_O_6_	6,7-Dimethoxy-7-epirosmanol
22.413	23.257	23.279	23.260	480.090	480.093	6.10	C_21_H_20_O_13_	Quercetin-3′-glucuronide
22.673	24.109	22.673	21.463	348.266	348.266	−0.54	C_22_H_36_O_3_	6-Pentadecyl salicylic acid
23.381	23.324	23.381	23.376	354.095	354.096	1.85	C_16_H_18_O_9_	Chlorogenic acid
23.535	ND	24.424	24.366	178.062	178.062	−3.86	C_10_H_10_O_3_	4-Methoxycinnamic acid
23.574	ND	ND	0.930	315.147	315.148	3.52	C_18_H_21_NO_4_	N-Dihydroferuloyltyramine
24.155	11.711	ND	17.840	330.183	330.185	5.89	C_20_H_26_O_4_	Carnosol
26.262	26.531	ND	ND	422.158	422.158	0.53	C_21_H_26_O_9_	(Dimethylallyl)scopoletin 7-glucoside
27.230	27.200	27.260	ND	370.090	370.092	5.39	C_16_H_18_O_10_	Isoferulic acid 3-O-glucuronide
29.956	29.956	30.097	30.067	801.224	801.223	−1.14	C_38_H_41_O_19_	Malvidin 3-(coumaroylglucoside)glucoside
30.636	ND	ND	ND	625.177	625.177	−0.51	C_28_H_33_O_16_	4′-O-Methyldelphinidin 3-O-rutinoside
34.776	ND	34.853	ND	834.216	834.214	−2.35	C_45_H_38_O_16_	Epifisetinidol-β-Epicatechin-β-Epifisetinidol
35.425	34.704	34.792	34.767	534.101	534.102	1.55	C_24_H_22_O_14_	Luteolin 5-(6′-malonylglucoside)
35.928	35.993	ND	ND	616.106	616.108	3.26	C_28_H_24_O_16_	Quercetin 3-(2-galloylglucoside)
ND	ND	39.443	39.562	632.101	632.102	1.30	C_28_H_24_O_17_	Ascorbylepigallocatechin 3-gallate
ND	ND	39.476	ND	332.074	332.076	3.62	C_13_H_16_O_10_	Glucogallic acid 4-O-glucoside
ND	ND	39.752	39.960	934.259	934.263	4.02	C_39_H_50_O_26_	Quercetin 3-sophorotrioside 7 rhamnoside
ND	ND	41.423	ND	770.191	770.194	4.45	C_33_H_38_O_21_	6-Hydroxyluteolin 6-glucoside-7-(hydroxy3-methylglutaryl) glucoside
48.548	ND	2.409	ND	576.127	576.125	−372	C_30_ H_24_O_12_	Epicatechin-β-catechin

Abbreviation: ND = not detected, ppm = part per million, T_R_ = retention time.

**Table 7:** Existence of tocopherols and tocotrienols in KLWE (A), KLEE (B), KFWE (C), and KFEE (D) assayed using UHPLC-ESI-QTOF-MS.

Time	Mass	Molecular	Possible Compound	Sample			
(min)	(*m*/*z*)	Formula		A	B	C	D
22.392	430.38	C_29_H_50_O_2_	α-Tocopherol				+
25.433	396.30	C_27_H_40_O_2_	δ-Tocotrienol				+
27.194	424.33	C_29_H_44_O_2_	α-Tocotrienol			+	+
30.264	410.31	C_28_H_42_O_2_	γ-Tocotrienol	+	+		
30.950	446.37	C_29_H_50_O_3_	α-Tocopherolquinone	+	+		
31.906	416.36	C_28_H_48_O_2_	β-Tocopherol	+	+	+	+
41.805	535.40	C_35_H_53_NO_3_	α-Tocopherol nicotinate		+		

+ = detected.

**Table 8:** Absolute values of C, EC and EGCG contents in the water and ethanolic extracts from kepel leaves and fruits, without and with PEF treatment.

Time (min)	KLWE (mg/g)	KLWE^ (mg/g)	KLEE (mg/g)	KLEE^ (mg/g)	KFWE (mg/g)	KFWE^ (mg/g)	KFEE (mg/g)	KFEE^ (mg/g)
8.24 ± 0.03	153.7	846.8	335.3	905.1	236.7	136.8	237.8	289.4
11.26 ± 0.20	338.2	921.4	245.0	616.9	135.4	118.2	147.6	224.2
13.37 ± 0.21	ND	799.9	ND	231.9	2892.2	ND	ND	ND

Symbol: ^ = PEF.

**Table 9:** Volatile organic compounds in kepel fruits processed by HS-SPME and ethanolic extraction and identified using GC-MS.

Peak	T_R_ (min)	Relative PA of TIC (%)	Identified Compound	Precursor [M^+^] (*m*/*z*)	Key MS/MS Fragments [M]^+^ (*m*/*z*)	Match (%)	MCL
1	8.85	0.891	α-Pinene	136	136, 121, 91	94	2a
2	10.67	0.991	β-Pinene	136	136, 107, 93, 69	97	2a
3	11.24	8.697	β-Myrcene	136	136, 93, 69, 41	70	2a
4	12.84	1.234	Limonene	136	136, 93, 68	97	2a
5	12.94	1.027	Eucalyptol	154	139, 111, 108, 95, 81, 71, 55, 43	98	2a
6	13.60	7.626	Ocimene	136	136, 93, 69, 41	98	2a
7	15.12	9.747	α-Terpinolene	136	136, 121	75	2a
8	15.76	1.438	Tricyclene	136	107, 93, 91, 79, 67, 41, 21	NR	2
9	26.91	0.393	α-Bergamotene	204	189, 161, 147, 133, 119, 93, 91, 69	98	2a
10	27.10	30.817	Trans β-Caryophyllene	204	161, 133, 119, 93, 69, 55, 41	82	2a
11	27.54	3.575	α-Bergamotene	204	189, 161, 147, 133, 119, 93, 91	91	2b
12	28.25	10.304	α-Humulene	204	204, 161, 93	99	2a
13	29.80	1.765	α-Farnesene	204	204, 161, 93, 81, 69	94	2b
14	30.39	0.456	β-Sesquiphellandrene	204	204, 161, 119, 105, 93, 41	NR	4
15	30.89	0.649	α-Bisabolene	204	204, 161, 119, 93	89	2a
16	31.50	0.693	Nerolidol B	222	204, 189, 161, 121, 93, 41	NR	4
17	32.54	4.216	β-Gurjunen	204	204, 161, 133, 119, 105, 93, 41	NR	4
18	33.28	4.255	α-Cadinene	204	204, 161, 133, 119, 105, 93, 41	NR	4
19	34.22	6.054	α-Eudesmol	222	223, 222, 204, 189, 161, 147, 119, 93, 41	NR	4
20	34.49	5.171	Bulnesol	222	223, 222, 204, 189, 161, 147, 119, 93, 41	NR	4

Abbreviation: NR = not reported, TIC = total ion count.

## Data Availability

The original contributions presented in this study are included in the article. Further inquiries can be directed to the corresponding author.

## Abbreviations/Symbols

The following abbreviations and symbols are used in this manuscript:

ANOVA	Analysis of Variance
bp	Base pair
C	Catechin
C	Content
DAD	Diode array detector
DI	Deionized water
DNA	Deoxyribonucleic acid
dNTPs	Deoxyribonucleotide triphosphates
EC	Epicatechin
EGCG	Epigallocatechin 3-gallate
ESI	Electrospray ionization
GA	Gallic acid
GAE	Gallic acid equivalent
GC	Gas chromatography
HPLC	High-performance liquid chromatography
HS	Headspace
ISSR	Inter sample sequence repeat
KFEE	Ethanolic extract of kepel fruits
KLEE	Ethanolic extract of kepel leaves
KFWE	Water extract of kepel fruits
KLWE	Water extract of kepel leaves
LOD	Limit of detection
LOQ	Limit of quantitation
M	Marker
MCL	Match Confidence Levels
MS	Mass spectrometry
*m*/*z*	Mass-to-charge ratio
*N*	Number
OD	Optical density
PCR	Polymerase chain reaction
PDA	Photodiode array
PEF, ^	Pulsed electric field
PTFE	Polytetrafluoroethylene
Q	Quercetin
QE	Quercetin equivalent
QTOF	Quadrupole time-of-flight
RNase A	Ribonuclease A
rpm	Revolution per minute
*S*. *burahol*	*Stelechocarpus burahol*
SCoT	Start codon targeted
SPME	Solid phase microextraction
TBE	Tris-borate-ethylenediamine tetraacetate
TFC	Total flavonoid content
TIC	Total ion count
T_m_	Melting temperature
TPC	Total phenolic content
trnL	Transfer RNA-leucine
UHPLC	Ultrahigh performance liquid chromatography
UV-Vis	Ultraviolet-visible
*w*/*v*	Weight by volume
°C	Degree Celsius
